# A Proving of Oil of Winter-green

**Published:** 1892-10

**Authors:** S. S. Simmons

**Affiliations:** Susquehanna, Pa.


### A PROVING OF OIL OF WINTERGREEN.

S. S. Simmons, Susquehanna, Pa.

Was called to see Mrs. W. at eleven P. M., and as I entered the house the husband said he had an Indian woman in the bed there, pointing to the bedroom. I went in and there found the reddest white woman I ever saw, and there she lay with her teeth chattering as though she had an ague chill, but said she was not cold but on the contrary burning up. Said I, "What have you been doing?" She replied, "A little past ten this evening I had a little stomach-ache and thought I would take two or three drops of wintergreen oil, but in dropping it in a spoon some ran out of the vial. I don't know how many drops, but I took it all, and in half an hour I was just as you now find me." Very red all over body with a fine rash; burning and stinging all over her, so she cannot lie still; teeth chattering and shaking so she could hardly talk; face swollen or puffed up with large sacks under the eyes. No pain, only the burning and stinging, and as red as a piece of red flannel. I could see the indications for but one remedy, and I gave it immediately. I gave Apis$^{M}$ one dose dry on tongue, and in about five minutes she stopped shaking, her teeth stopped chattering, and she said she felt so queer as if she was going off, and in a few minutes she rolled on to her right side and seemed unconscious for about two minutes, then looked up and said she felt better. The burning and stinging was all gone, but she still felt a prickling of skin, which soon passed off. She then said she felt all right again. I left another powder of Apis, with instructions not to take it as long as she continued to feel better. The next morning she was able to get breakfast and eat it, too. She said she had learned a good lesson and should take no more remedies unless I prescribed them. So much for following the teachings of true Homœopathy.

